# Symmetry‐Breaking Charge‐Transfer Chromophore Interactions Supported by Carbon Nanodots†

**DOI:** 10.1002/anie.202004638

**Published:** 2020-05-20

**Authors:** Michele Cacioppo, Tobias Scharl, Luka Đorđević, Alejandro Cadranel, Francesca Arcudi, Dirk M. Guldi, Maurizio Prato

**Affiliations:** ^1^ Department of Chemical and Pharmaceutical Sciences University of Trieste, and INSTM, unit of Trieste Via Licio Giorgieri 1 34127 Trieste Italy; ^2^ Department of Chemistry and Pharmacy, Interdisciplinary Center for Molecular Materials Friedrich-Alexander Universität Erlangen-Nürnberg Egerlandstrasse 3 91058 Erlangen Germany; ^3^ Present address: Department of Chemistry Northwestern University 2145 Sheridan Road Evanston IL 60208 USA; ^4^ Present address: Simpson Querrey Institute Northwestern University 303 E. Superior Chicago IL 60611 USA; ^5^ Universidad de Buenos Aires Facultad de Ciencias Exactas y Naturales Departamento de Química Inorgánica, Analítica y Química Física Pabellón 2, Ciudad Universitaria C1428EHA Buenos Aires Argentina; ^6^ CONICET— Universidad de Buenos Aires Instituto de Química-Física de Materiales, Medio Ambiente y Energía (INQUIMAE) Pabellón 2, Ciudad Universitaria C1428EHA Buenos Aires Argentina; ^7^ Center for Cooperative Research in Biomaterials (CIC biomaGUNE) Basque Research and Technology Alliance (BRTA) Paseo de Miramon 182 20014 Donostia San Sebastián Spain; ^8^ Basque Foundation for Science Ikerbasque Bilbao 48013 Spain

**Keywords:** carbon dots, charge transfer, chromophores, phthalocyanines, symmetry breaking

## Abstract

Carbon dots (CDs) and their derivatives are useful platforms for studying electron‐donor/acceptor interactions and dynamics therein. Herein, we couple amorphous CDs with phthalocyanines (Pcs) that act as electron donors with a large extended π‐surface and intense absorption across the visible range of the solar spectrum. Investigations of the intercomponent interactions by means of steady‐state and pump‐probe transient absorption spectroscopy reveal symmetry‐breaking charge transfer/separation and recombination dynamics within pairs of phthalocyanines. The CDs facilitate the electronic interactions between the phthalocyanines. Thus, our findings suggest that CDs could be used to support electronic couplings in multichromophoric systems and further increase their applicability in organic electronics, photonics, and artificial photosynthesis.

## Graphical abstract

**Close encounters**: Carbon nanodots have been functionalized with phthalocyanine chromophores by a covalent synthetic approach. This approach made possible the formation of symmetry‐breaking charge transfer/separation states between two or more chromophore units connected on the carbon dot surface.

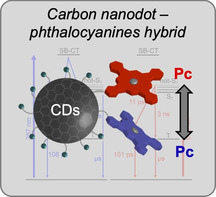

Multichromophoric systems play an important role in both organic electronics and photosynthetic light harvesting, with the spatial arrangement of the chromophores governing their electronic communication.^1^ For example, in the photosynthetic reaction center of purple bacteria, electron transfer from a bacteriochlorophyll “special pair”, surrounded by two branches of protein‐bound cofactors, is preceded by a symmetry‐breaking charge‐transfer (SB‐CT) state; this is generally defined as arising from a photoexcited process, where a pair of identical chromophores produces a desymmetrized charge‐transfer (CT) state. This ultrafast‐formed state governs charge separation in photosystem II.^2^ Gathering a comprehensive understanding is vital for designing and preparing efficient organic electronic devices. Molecular materials which are capable of undergoing SB‐CT will very likely find applications in fields such as organic photovoltaics,^3^ photonics,^4^ and artificial photosynthesis.^5^

In recent years, there have been many efforts to study interactions between chromophores in nonbiological molecular assemblies. Most notable are pairs of arenes,^6^ perylenes,^7^ perylene diimides,^8^ boron dipyrromethenes/dipyridylmethenes,^9^ and metallodipyrrins,^3^, ^10^ to name just a few. In terms of effective interchromophore communication, the spacer between them is one of the most critical variables. For example, zinc porphyrins have been linked through phenylene(s),^11^ naphthalene,^11^, ^12^ phenanthrene,^12^ hexaphenylbenzene,^13^ hexa‐*peri*‐hexabenzocoronene bridges,^14^ as well as well‐defined nanographene spacers.^15^ Recently, porphyrins, which were linked through hexa‐*peri*‐hexabenzocoronene (HBC) spacers, were shown to interact through the π‐surface of the HBC. The most compelling evidence for this is a distinct split of the porphyrin Soret‐band absorption.14a

In the context of photoactive chromophore assemblies, carbon dots (CDs) have been attracting attention.^16^ These small (<10 nm) carbon‐based nanoparticles have been employed, both as electron donor (D) and acceptor (A), in electron‐donor/acceptor systems based on covalent or noncovalent approaches.^17^ Notably our groups have previously investigated amorphous nitrogen‐doped CDs (referred to as *a*‐N‐CDs or nitrogen‐doped carbon nanodots, NCNDs), and their electron‐transfer interactions with a *meso*‐tetraarylporphyrin (H_2_P).17a It was found that the covalent NCND‐H_2_P conjugate, immediately after photoexcitation with visible light, undergoes charge separation to form the one‐electron‐oxidized form of H_2_P and the one‐electron‐reduced form of NCND. We now turn our attention to phthalocyanines (Pcs), well‐known electron donors, which possess unique properties such as good thermal and (photo)chemical stabilities, as well as intense absorptions in the visible range.^18^ Our interest stems also from some previous reports of Pc blends with different CDs, namely, graphitized carbon dots (discoidal and quasi‐spherical in shape).^19^ These blends were used to improve the absorption of visible light,19a to enhance electron‐transfer/transport characteristics in inorganic quantum dots solar cells,19b and to enhance the nonlinear optical response of the nanomaterials.19d It is important to note that no symmetry‐breaking charge transfer (SB‐CT)/separation (SB‐CS) has been observed in any of the aforementioned CD‐based D‐A materials.

Herein, we report the preparation of a covalent electron‐donor/acceptor conjugate consisting of NCNDs and a zinc phthalocyanine (ZnPc). Notably, we show that, upon absorption of light at λ=387 nm, the NCNDs carrying the ZnPcs undergo a SB‐CT (ZnPc^δ−^‐ZnPc^δ+^), which is followed by a SB‐CS to afford (ZnPc^−^‐ZnPc^+^). Similarly, after photoexcitation at λ=675 nm, a SB‐CS is also observed, which, in this particular case, evolved from a hot‐S_1_ state. Taken in concert, the results show that NCNDs provide a scaffold to spatially arrange ZnPcs and facilitate the electronic communication between two or more of them.

The NCND‐ZnPc conjugate (Figure 1) was prepared by a carbodiimide (EDC) mediated coupling of the amino groups of the NCNDs^20^ and the carboxyphthalocyanine (ZnPc);^21^ the characterizations of the conjugate were in accordance with the literature (see the Materials Section in the Supporting Information). Following the reaction and the removal of DMF, the reaction mixture was purified by size‐exclusion chromatography (SEC, Sephadex LH‐20 resin in methanol). The first dark blue band, corresponding to the NCND‐ZnPc conjugate, was collected, the organic solvent was removed under reduced pressure, and the final blue solid was obtained after freeze‐drying an aqueous solution. The successful formation of the NCND‐ZnPc conjugate was apparent as soon as it was dissolved in methanol, as it had a noticeable difference in solubility in polar organic solvents compared to the starting carboxyphthalocyanine. Furthermore, from a Kaiser test, which was performed with NCNDs and NCND‐ZnPc, we estimated the presence of a lower amount of free amino groups (100 μmol g^−1^) on the nanoparticle surface, compared to pristine NCNDs (1350 μmol g^−1^). Additional characterization by FTIR spectrophotometry (KBr) confirmed the formation of the amide bond (Figure S1). Specifically, in the conjugate we observe the absence of the ZnPc carboxylic band at $\tilde{\nu }$ =1716 cm^−1^, the presence of the ZnPc signature bands in the form of C−H stretching at $\tilde{\nu }$ =2961, 2928, and 2850 cm^−1^ as well as C−H bending at $\tilde{\nu }$ =1380 cm^−1^, and the appearance of C=O and N−H bending signals of amide bands at $\tilde{\nu }$ =1637 and 1565 cm^−1^, respectively. The composition was further probed by X‐ray photoelectron spectroscopy (XPS; Figure S2). The survey spectrum of the hybrid material shows the three peaks corresponding to the C 1s, N 1s, and O 1s species (the atomic percentages for C, N, O are 84.3, 8.0 and 7.7) and the Zn 2p_3/2_ species is visible in the high‐resolution spectrum. The higher C/N ratio observed for the hybrid (C/N=10.5) compared to the NCNDs (C/N=4.25)20a is consistent with the presence of the organic macrocycle (deconvoluted spectra are shown in Figure S3). The N 1s spectrum also provides evidence of functionalization (Figure S3a,b). It can be deconvoluted into surface components observed for the two single entities,20a but the percentage of primary amine is lower compared to the NCNDs alone, in agreement with the decreased value of the Kaiser test.

**Figure 1:**
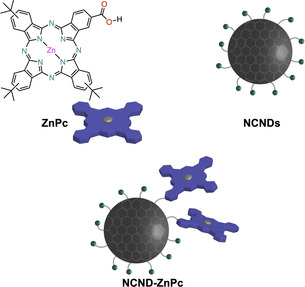
Schematic representation of the ZnPc reference, NCND reference, and NCND‐ZnPc conjugate discussed here. NCNDs are represented as an amorphous carbonaceous core (gray) and an amino‐rich surface (green color).

Thermogravimetric analyses were used to compare the weight residues (or loss) of the hybrid and reference materials (Figure S4). The residual weight at 450 °C for the NCND‐ZnPc and the NCND alone are 50 % and 14 %, respectively. Taking into account that zinc phthalocyanines have good thermal stability (>90 % residual weight at 450 °C),^22^ we conclude that the lower residual loss from the conjugate is due to the presence of the ZnPc, and it can be roughly calculated that the latter constitutes about half of the material. Finally, morphological characterization by atomic force microscopy (AFM) showed the presence of larger nanoparticles (4.19±1.11 nm), when compared to pristine NCNDs20a (Figure S5).

The optoelectronic properties of the NCND‐ZnPc conjugate were first analyzed by comparison with the two references, namely NCNDs and ZnPc, using steady‐state absorption and fluorescence spectroscopy (Figure 2, methanol at room temperature).

**Figure 2:**
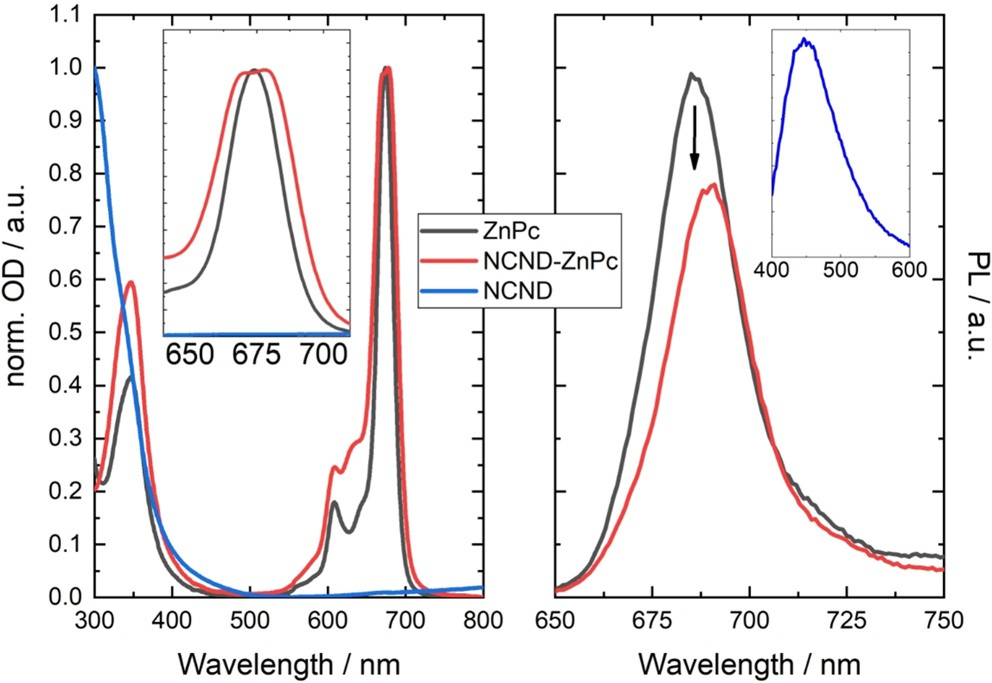
Absorption and fluorescence spectra. Left: Absorption spectra of the NCND reference (blue trace), the ZnPc reference (black trace), and the NCND‐ZnPc conjugate (red trace) in methanol at room temperature (the inset shows an enlargement of the Q‐band absorption). Right: Fluorescence spectra of the ZnPc reference (black trace) and the NCND‐ZnPc conjugate (red trace) with matching absorbance at an excitation wavelength of λ=387 nm in methanol and at room temperature (the inset shows the NCND fluorescence upon excitation at λ=387 nm in methanol and at room temperature).

The absorption spectrum of ZnPc shows the typical Q‐band absorption at λ=674 nm and a weaker Soret‐band absorption at λ=346 nm. The NCNDs, on the other hand, show the typical absorption spectrum with a broad signal that tails into the visible region. The NCND‐ZnPc conjugate presents a different absorption spectrum than the two references. In particular, the Q‐band absorptions split into two maxima of similar intensity at λ=678 and 670 nm (Figure 2 left, inset). This phenomenon, known as Davydov splitting,^23^ is associated with electronic communication between two or more ZnPc units that are in proximity to each other.14a, 24 It is noteworthy that Davydov splitting is not observed in molecular aggregates of ZnPc, since in the latter case a new band appears at λ=640 nm (Figure S6). The NCND‐ZnPcs were additionally studied in the excited state and, again, compared to the references (Figure 2, right). To excite ZnPc, an excitation wavelength of λ=387 nm (3.2 eV) was used and the samples were adjusted to obtain the same optical density. ZnPc shows a strong fluorescence at λ=686 nm, with a quantum yield of 0.15. In the NCND‐ZnPc conjugate, a fluorescence quenching of 22 % is observed, together with a 4 nm red‐shift to λ=690 nm. An analogous red shift is seen, with a lower fluorescence quenching of 9 %, on using an excitation wavelength of λ=675 nm (1.84 eV; Figure S7). The difference in the fluorescence quenching efficiency is due to the type of electronic transition which is excited in each particular case (see below). These findings suggest that symmetry‐breaking interactions supported by the NCNDs promote additional decay pathways upon excitation.

This aspect was further investigated by electrochemical means (Figure 3). For the NCND reference, rather weak and broad signals, which are hard to dissect, were recorded. The other reference, ZnPc, shows oxidations at +0.59 and +0.82 V versus Ag/AgCl and reductions at −0.73 and −0.85 V versus Ag/AgCl. NCND‐ZnPc, in contrast, showed three (rather than two) oxidations and reductions at +0.55, +0.72, +0.84 V and −0.78, −0.90, −1.01 V, respectively. This result strongly suggests that, when coupled, the ZnPc‐centered orbitals are split because of ground‐state electronic interactions with another ZnPc, which is facilitated by the NCNDs. For this reason, a SB‐CS state is expected at around 1.33 eV above the ground state.

**Figure 3:**
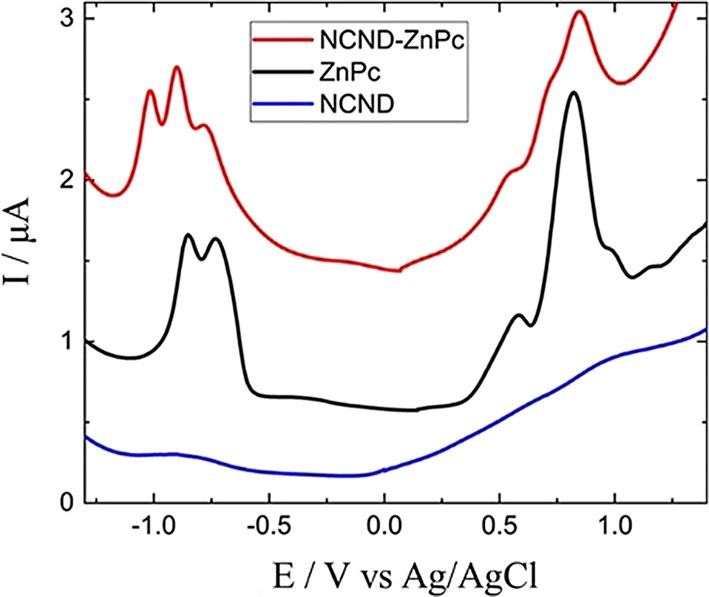
Electrochemistry of the NCND reference (blue trace), ZnPc reference (black trace), and the NCND‐ZnPc conjugate (red trace) in methanol at room temperature.

To better understand the excited‐state dynamics responsible for the ZnPc fluorescence quenching, femtosecond time‐resolved transient absorption spectroscopy (fsTAS) experiments were performed. These were done for the conjugate and the ZnPc reference, with excitation at λ=387 nm in methanol and at room temperature. The ZnPc reference (Figure S8) showed positive transients at λ=485, 590, 630, and 808 nm, next to a negative signal at λ=674 nm. These features persist to the nanosecond timescale, after which a positive transient at λ=480 nm and negative ones at λ=609 and 674 nm appear. The global analysis suggests a three‐species kinetic model with two exponentially decaying species with time constants of 7 ps and 2.2 ns, together with an infinitely lived species. These two decays are ascribed to an internal conversion S_1_←S_2_ and an intersystem crossing T_1_←S_1_. The triplet (T_1_) excited states were much longer‐lived with respect to the resolution of the experiments, which in turn helps to rationalize their infinitively lived nature.^25^ Similar results were obtained when the ZnPc was excited at λ=675 nm (Figure S9). The excitation leads to a hot‐S_1_ population that decays in 1.6 ps to afford S_1_. Then, the intersystem crossing T_1_←S_1_ takes 2.9 ns, preceding the decay to the ground state that occurs on the microsecond timescale.

Compared to ZnPc, NCND‐ZnPc shows very different excited state dynamics. Excitation at λ=387 nm leads to two minima at λ=633 and 670 nm (Figure 4). These signals are accompanied by positive transients at λ=547 and 750 nm, which, importantly, were not seen in the ZnPc reference. Moreover, these findings suggest the typical fingerprint absorption of the one‐electron‐oxidized ZnPc.^26^ In contrast, on the nanosecond timescale, the transient absorption spectra resemble those observed for the ZnPc reference. The kinetic model which is derived from these data involves three exponentially decaying species and a fourth one, which is persistent on the timescale of these experiments.

**Figure 4:**
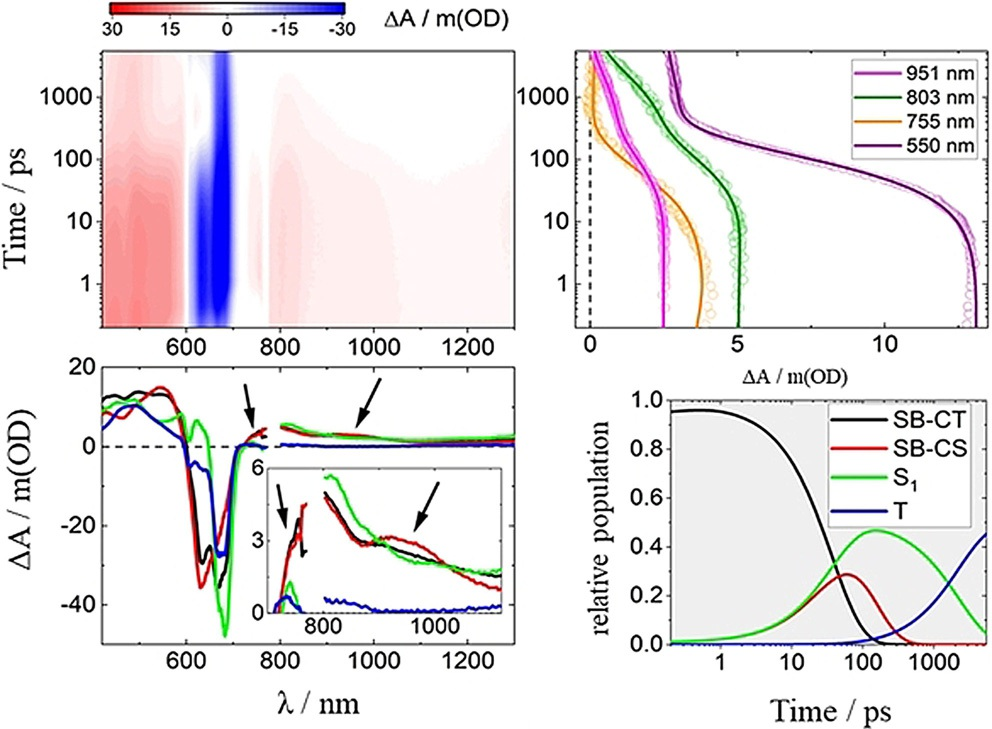
Top left: Differential absorption 3D map obtained upon fsTAS of NCND‐ZnPc in methanol at room temperature upon excitation at λ=387 nm. Top right: time absorption profiles and corresponding line fittings at λ=951 (magenta), 803 (dark green), 755 (orange), and 550 nm (purple). Bottom left: species‐associated differential spectra of the SB‐CT (black), SB‐CS (red), S_1_ (green), and T_1_ (blue) excited states. Bottom right: concentration evolution over time.

Taking also into account the results of fluorescence spectroscopy, a parallel target model was necessary to fit the transient absorption measurements. This model considers two parallel cascades. One of the two cascades, responsible for the fluorescence, includes the same singlet excited state.^27^ This intersystem crossing occurs in 2.2 ns to afford the corresponding microsecond‐lived triplet excited state that was seen in the ZnPc reference (Figure 5). The second cascade is related to the fluorescence quenching and it is characterized by two excited states with distinct transients at λ=547, 750, and 925 nm. Notably, the maxima at λ=547 and 750 nm match the ones responsible for the observed formation of one‐electron‐oxidized ZnPc.^26^ The NCNDs in contrast are characterized by a rather broad and featureless band, which extends all across the visible range of the solar spectrum. Moreover, the one‐electron‐reduced ZnPc shows a maximum at λ=1000 nm^28^ that resembles the one recorded for the conjugate. This global information suggests a system formed from a NCND nanoparticle that binds at least two ZnPcs. Here, a SB‐CT/SB‐CS occurs, by oxidation of one ZnPc moiety, on one hand, and a reduction of another ZnPc moiety, on the other hand. Thus, these two species that are involved in the fluorescence quenching could be assigned in one case to a symmetry‐breaking charge‐transfer ZnPc^δ−^‐ZnPc^δ+^ state (SB‐CT), which is populated directly upon the absorption of light. In the other case, the cascade could involve the symmetry‐breaking charge‐separated ZnPc^−^‐ZnPc^+^ state (SB‐CS), which is formed in 37 ps and reinstates the ground state through charge recombination in 108 ps (Figure 5). When excited at λ=675 nm, the excited‐state dynamics of NCND‐ZnPc are quite similar (Figure S10). More specifically, although the SB‐CS state evolves from a hot‐S_1_ state in 11 ps, the charge recombination takes 101 ps, and matches the results obtained on exciting at λ=387 nm (Figure 5). To further compare these states, Figure S11 shows the differential spectra of the NCND‐ZnPc generated species upon excitation at λ=387 and 675 nm.

**Figure 5:**
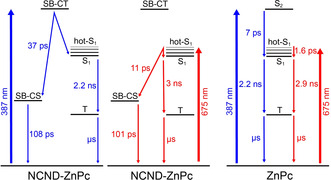
Target models employed to fit the fsTAS data. Left: NCND‐ZnPc conjugate under excitation at λ=387 nm. Center: NCND‐ZnPc conjugate under excitation at λ=675 nm. Right: ZnPc reference under excitation at λ=387 and 675 nm.

In summary, we have prepared a covalent conjugate between N‐doped carbon nanodots (NCNDs) and zinc phthalocyanines (ZnPcs). Symmetry‐breaking (SB) interactions within a ZnPc pair were evident in the absorption profile of the NCND‐ZnPc conjugate, which shows the Q‐band absorptions presenting Davydov splitting into two maxima of similar intensity. Notably, steady‐state fluorescence experiments show quenching in the conjugate, accompanied by a red‐shift of the fluorescence maximum, on excitation at both λ=387 and 687 nm. This electronic communication, between at least two ZnPc species close to one another on the NCNDs, was confirmed also by electrochemical experiments. Further investigations, by means of steady‐state and pump‐probe transient absorption spectroscopy, revealed the SB‐CT/SB‐CS and recombination dynamics in the hybrid material. The present system, therefore, differs from the CND‐H_2_P conjugate, which lacked any evidence of close contacts between the H_2_Ps on the periphery of the NCNDs.

We have thus provided further evidence of the beneficial integration of CDs into electron‐donor/acceptor assemblies, this time by performing a new role as a scaffold. Although precise control of the number and position of the chromophores coupled on the CD surface remains beyond reach, the results presented herein should stimulate further research in CD‐based D‐A systems and applications in solar‐energy conversion. For example, the use of CDs as scaffolds for chromophores of a different nature, as well as using CDs with different structures is envisaged.

## Conflict of interest

The authors declare no conflict of interest.

## Supporting information

As a service to our authors and readers, this journal provides supporting information supplied by the authors. Such materials are peer reviewed and may be re‐organized for online delivery, but are not copy‐edited or typeset. Technical support issues arising from supporting information (other than missing files) should be addressed to the authors.

Supplementary

## References

[1a] D. Gust , T. A. Moore , A. L. Moore , Acc. Chem. Res. 2001, 34, 40–48; 11170355 10.1021/ar9801301

[1b] D. Holten , D. F. Bocian , J. S. Lindsey , Acc. Chem. Res. 2002, 35, 57–69; 11790089 10.1021/ar970264z

[1c] M. R. Wasielewski , Acc. Chem. Res. 2009, 42, 1910–1921; 19803479 10.1021/ar9001735

[1d] N. Aratani , D. Kim , A. Osuka , Acc. Chem. Res. 2009, 42, 1922–1934; 19842697 10.1021/ar9001697

[1e] R. Bhosale , J. Míšek , N. Sakai , S. Matile , Chem. Soc. Rev. 2010, 39, 138–149; 20023844 10.1039/b906115k

[1f] F. D'Souza , O. Ito , Chem. Soc. Rev. 2012, 41, 86–96; 21975532 10.1039/c1cs15201g

[1g] A. Saeki , Y. Koizumi , T. Aida , S. Seki , Acc. Chem. Res. 2012, 45, 1193–1202; 22676381 10.1021/ar200283b

[1h] S. Sengupta , F. Würthner , Acc. Chem. Res. 2013, 46, 2498–2512. 23865851 10.1021/ar400017u

[2a] S. R. Meech , A. J. Hoff , D. A. Wiersma , Chem. Phys. Lett. 1985, 121, 287–292;

[2b] D. J. Lockhart , S. G. Boxer , Biochemistry 1987, 26, 664–668;

[2c] E. J. P. Lathrop , R. A. Friesner , J. Phys. Chem. 1994, 98, 3056–3066;

[2d] K. N. Ferreira , T. M. Iverson , K. Maghlaoui , J. Barber , S. Iwata , Science 2004, 303, 1831–1838. 14764885 10.1126/science.1093087

[3] A. N. Bartynski , M. Gruber , S. Das , S. Rangan , S. Mollinger , C. Trinh , S. E. Bradforth , K. Vandewal , A. Salleo , R. A. Bartynski , W. Bruetting , M. E. Thompson , J. Am. Chem. Soc. 2015, 137, 5397–5405. 25826321 10.1021/jacs.5b00146

[4] E. Collet , M.-H. Lemée-Cailleau , M. Buron-Le Cointe , H. Cailleau , M. Wulff , T. Luty , S.-Y. Koshihara , M. Meyer , L. Toupet , P. Rabiller , S. Techert , Science 2003, 300, 612–615. 12714737 10.1126/science.1082001

[5a] B. Rybtchinski , L. E. Sinks , M. R. Wasielewski , J. Am. Chem. Soc. 2004, 126, 12268–12269; 15453751 10.1021/ja0460514

[5b] A. L. Sisson , N. Sakai , N. Banerji , A. Fürstenberg , E. Vauthey , S. Matile , Angew. Chem. Int. Ed. 2008, 47, 3727–3729; 10.1002/anie.200800203 18366052

[6a] H. Lueck , M. W. Windsor , W. Rettig , J. Phys. Chem. 1990, 94, 4550–4559;

[6b] F. C. Grozema , M. Swart , R. W. J. Zijlstra , J. J. Piet , L. D. A. Siebbeles , P. T. van Duijnen , J. Am. Chem. Soc. 2005, 127, 11019–11028. 16076209 10.1021/ja051729g

[7a] V. Markovic , D. Villamaina , I. Barabanov , L. M. Lawson Daku , E. Vauthey , Angew. Chem. Int. Ed. 2011, 50, 7596–7598; 10.1002/anie.201102601 21728220

[7b] A. Aster , G. Licari , F. Zinna , E. Brun , T. Kumpulainen , E. Tajkhorshid , J. Lacour , E. Vauthey , Chem. Sci. 2019, 10, 10629–10639. 10.1039/c9sc03913a PMC8133027 34040711

[8a] M. W. Holman , P. Yan , D. M. Adams , S. Westenhoff , C. Silva , J. Phys. Chem. A 2005, 109, 8548–8552; 16834252 10.1021/jp0502050

[8b] Y. Wu , R. M. Young , M. Frasconi , S. T. Schneebeli , P. Spenst , D. M. Gardner , K. E. Brown , F. Würthner , J. F. Stoddart , M. R. Wasielewski , J. Am. Chem. Soc. 2015, 137, 13236–13239; 26418462 10.1021/jacs.5b08386

[8c] J. Sung , A. Nowak-Krol , F. Schlosser , B. Fimmel , W. Kim , D. Kim , F. Wurthner , J. Am. Chem. Soc. 2016, 138, 9029–9032. 27407012 10.1021/jacs.6b04591

[9a] M. T. Whited , N. M. Patel , S. T. Roberts , K. Allen , P. I. Djurovich , S. E. Bradforth , M. E. Thompson , Chem. Commun. 2012, 48, 284–286; 10.1039/c1cc12260f 22105418

[9b] J. H. Golden , L. Estergreen , T. Porter , A. C. Tadle , D. M. R. Sylvinson , J. W. Facendola , C. P. Kubiak , S. E. Bradforth , M. E. Thompson , ACS Appl. Energy Mater. 2018, 1, 1083–1095.

[10] C. Trinh , K. Kirlikovali , S. Das , M. E. Ener , H. B. Gray , P. Djurovich , S. E. Bradforth , M. E. Thompson , J. Phys. Chem. C 2014, 118, 21834–21845. 10.1021/jp506855t PMC4174994 25270268

[11] S. Cho , M. C. Yoon , C. H. Kim , N. Aratani , G. Mori , T. Joo , A. Osuka , D. Kim , J. Phys. Chem. C 2007, 111, 14881–14888.

[12] N. Toyama , M. Asano-Someda , T. Ichino , Y. Kaizu , J. Phys. Chem. A 2000, 104, 4857–4865.

[13a] M. Takase , R. Ismael , R. Murakami , M. Ikeda , D. Kim , H. Shinmori , H. Furuta , A. Osuka , Tetrahedron Lett. 2002, 43, 5157–5159;

[13b] H. S. Cho , H. Rhee , J. K. Song , C.-K. Min , M. Takase , N. Aratani , S. Cho , A. Osuka , T. Joo , D. Kim , J. Am. Chem. Soc. 2003, 125, 5849–5860; 12733926 10.1021/ja021476g

[13c] M. M. Martin , M. Dill , J. Langer , N. Jux , J. Org. Chem. 2019, 84, 1489–1499. 30596243 10.1021/acs.joc.8b02907

[14a] M. M. Martin , D. Lungerich , P. Haines , F. Hampel , N. Jux , Angew. Chem. Int. Ed. 2019, 58, 8932–8937; 10.1002/anie.201903654 30968516

[14b] M. M. Martin , D. Lungerich , F. Hampel , J. Langer , T. K. Ronson , N. Jux , Chem. Eur. J. 2019, 25, 15083–15090. 31429504 10.1002/chem.201903113 PMC6899994

[15] T. Umeyama , T. Hanaoka , H. Yamada , Y. Namura , S. Mizuno , T. Ohara , J. Baek , J. Park , Y. Takano , K. Stranius , N. V. Tkachenko , H. Imahori , Chem. Sci. 2019, 10, 6642–6650. 31367317 10.1039/c9sc01538h PMC6624990

[16a] F. Arcudi , L. Đorđević , M. Prato , Acc. Chem. Res. 2019, 52, 2070–2079; 31335113 10.1021/acs.accounts.9b00249

[16b] A. Cadranel , J. T. Margraf , V. Strauss , T. Clark , D. M. Guldi , Acc. Chem. Res. 2019, 52, 955–963; 30882201 10.1021/acs.accounts.8b00673

[16c] Z. Kang , S.-T. Lee , Nanoscale 2019, 11, 19214–19224; 31513215 10.1039/c9nr05647e

[16d] C. Xia , S. Zhu , T. Feng , M. Yang , B. Yang , Adv. Sci. 2019, 6, 1901316. 10.1002/advs.201901316 PMC6891914 31832313

[17a] F. Arcudi , V. Strauss , L. Đorđević , A. Cadranel , D. M. Guldi , M. Prato , Angew. Chem. Int. Ed. 2017, 56, 12097–12101; 10.1002/anie.201704544 28749592

[17b] K. H. Koh , S. H. Noh , T.-H. Kim , W. J. Lee , S.-C. Yi , T. H. Han , RSC Adv. 2017, 7, 26113–26119;

[17c] A. Ferrer-Ruiz , T. Scharl , P. Haines , L. Rodríguez-Pérez , A. Cadranel , M. Á. Herranz , D. M. Guldi , N. Martín , Angew. Chem. Int. Ed. 2018, 57, 1001–1005; 10.1002/anie.201709561 29193715

[17d] T. Scharl , A. Cadranel , P. Haines , V. Strauss , S. Bernhardt , S. Vela , C. Atienza , F. Gröhn , N. Martín , D. M. Guldi , Chem. Commun. 2018, 54, 11642–11644; 10.1039/c8cc05069d 30272065

[17e] A. Cadranel , V. Strauss , J. T. Margraf , K. A. Winterfeld , C. Vogl , L. Đorđević , F. Arcudi , H. Hoelzel , N. Jux , M. Prato , D. M. Guldi , J. Am. Chem. Soc. 2018, 140, 904–907; 29281276 10.1021/jacs.7b12434

[17f] L. Vallan , R. Canton-Vitoria , H. B. Gobeze , Y. Jang , R. Arenal , A. M. Benito , W. K. Maser , F. D'Souza , N. Tagmatarchis , J. Am. Chem. Soc. 2018, 140, 13488–13496; 30222336 10.1021/jacs.8b09204

[17g] L. Ðorđević , F. Arcudi , A. D'Urso , M. Cacioppo , N. Micali , T. Bürgi , R. Purrello , M. Prato , Nat. Commun. 2018, 9, 3442; 30143608 10.1038/s41467-018-05561-2 PMC6109168

[17h] T. Scharl , A. Ferrer-Ruiz , A. Saura-Sanmartín , L. Rodríguez-Pérez , M. Ángeles Herranz , N. Martín , D. M. Guldi , Chem. Commun. 2019, 55, 3223–3226; 10.1039/c8cc09990a 30806381

[17i] I. Srivastava , J. S. Khamo , S. Pandit , P. Fathi , X. Huang , A. Cao , R. T. Haasch , S. Nie , K. Zhang , D. Pan , Adv. Funct. Mater. 2019, 29, 1902466.

[18a] Phthalocyanines, Properties and Applications (Eds.: C. C. Leznoff, A. B. P. Lever), VCH, Weinheim, 1993;

[18b] Phthalocyanine Materials: Synthesis, Structure and Function (Ed.: N. B. McKeown), Cambridge University Press, Cambridge, 1998;

[18c] T. Inabe , H. Tajima , Chem. Rev. 2004, 104, 5503–5534; 15535658 10.1021/cr030649x

[18d] G. de la Torre , C. G. Claessens , T. Torres , Chem. Commun. 2007, 2000–2015; 10.1039/b614234f 17713062

[18e] G. de la Torre , G. Bottari , M. Sekita , A. Hausmann , D. M. Guldi , T. Torres , Chem. Soc. Rev. 2013, 42, 8049–8105; 23832123 10.1039/c3cs60140d

[18f] L. Martín-Gomis , F. Fernández-Lázaro , Á. Sastre-Santos , J. Mater. Chem. A 2014, 2, 15672–15682;

[18g] G. Bottari , G. de la Torre , T. Torres , Acc. Chem. Res. 2015, 48, 900–910. 25837299 10.1021/ar5004384

[19a] S. Hu , R. Tian , Y. Dong , J. Yang , J. Liu , S. Cao , RSC Adv. 2013, 3, 21447–21452;

[19b] R. Narayanan , M. Deepa , A. K. Srivastava , J. Mater. Chem. A 2013, 1, 3907–3918;

[19c] M. K. Barman , B. Jana , S. Bhattacharyya , A. Patra , J. Phys. Chem. C 2014, 118, 20034–20041;

[19d] N. Nwaji , O. J. Achadu , T. Nyokong , New J. Chem. 2018, 42, 6040–6050.

[20a] F. Arcudi , L. Đorđević , M. Prato , Angew. Chem. Int. Ed. 2016, 55, 2107–2112; 10.1002/anie.201510158 26733058

[20b] L. Ðorđević , F. Arcudi , M. Prato , Nat. Protoc. 2019, 14, 2931–2953. 31534230 10.1038/s41596-019-0207-x

[21] J.-J. Cid , J.-H. Yum , S.-R. Jang , M. K. Nazeeruddin , E. Martínez-Ferrero , E. Palomares , J. Ko , M. Grätzel , T. Torres , Angew. Chem. Int. Ed. 2007, 46, 8358–8362; 10.1002/anie.200703106 17912726

[22] N. He , Y. Chen , J. Bai , J. Wang , W. J. Blau , J. Zhu , J. Phys. Chem. C 2009, 113, 13029–13035.

[23] A. S. Davydov , Sov. Phys. Usp. 1964, 7, 145–178.

[24] J. J. Pie , P. N. Taylor , B. R. Wegewijs , H. L. Anderson , A. Osuka , J. M. Warman , J. Phys. Chem. B 2001, 105, 97–104.

[25] L. Wibmer , L. M. O. Lourenço , A. Roth , G. Katsukis , M. G. P. M. S. Neves , J. A. S. Cavaleiro , J. P. C. Tomé , T. Torres , D. M. Guldi , Nanoscale 2015, 7, 5674–5682. 25740090 10.1039/c4nr05719h

[26] Handbook of Porphyrin Science, Vol. 9 (Eds.: K. M. Kadish, K. M. Smith, R. Guilard), World Scientific, Singapore, 2010.

[27] Please note that the fluorescence of S_1_ in the nanoconjugate differs somewhat from that of just ZnPc. In turn, the relative population considered for S_1_ in NCND-ZnPc might not exactly reflect the 22 % fluorescence quenching.

[28a] P. C. Minor , M. Gouterman , A. B. P. Lever , Inorg. Chem. 1985, 24, 1894–1900;

[28b] D. W. Clack , J. R. Yandle , Inorg. Chem. 1972, 11, 1738–1742.

