# Supplementary material for: Symmetry‐Breaking Charge‐Transfer Chromophore Interactions Supported by Carbon Nanodots
Source: Angew Chem Int Ed Engl. 2020 May 20;59(31):12779–84. doi: 10.1002/anie.202004638 (PMC7496469; doi:10.1002/anie.202004638)
Supplement: Supplementary file 1 — Supplementary [file ANIE-59-12779-s001.pdf]

Angewandte Chemie
Eine Zeitschrift der Gesellschaft Deutscher Chemiker

Supporting Information

# Symmetry-Breaking Charge-Transfer Chromophore Interactions Supported by Carbon Nanodots**

*Michele Cacioppo[+], Tobias Scharl[+], Luka Đorđević,* Alejandro Cadranel, Francesca Arcudi, Dirk M. Guldi,* and Maurizio Prato**

anie_202004638_sm_miscellaneous_information.pdf

## Table of Contents

1. Materials ........................................................................................................ 1
2. Apparatus and Characterization ..................................................................... 1
3. Synthesis of NCND-ZnPc ................................................................................ 3
4. Supporting Figures.......................................................................................... 3
5. References........................................................................................................ 9

## 1. Materials

NCNDs were synthetized as previously reported.[1, 2] Ultrapure water was obtained by a MilliQ™ water purification system (water resistivity of 18.2 MΩ at 25 °C). All the solvent and reagents (reagent grade) were bought from Sigma-Aldrich and used without further purification.

ZnPc was synthetized by using 4-*tert*-butylphthalonitrile (32703-80-3, Sigma 423122-1G) and 1,2-dicyano-4-(methoxycarbonyl)benzene as precursors. 1,2-Dicyano-4-(methoxycarbonyl)benzene was prepared by two steps: (1) bromination with $Br_2$ in $H_2SO_4$ (0 °C) of methyl 4-bromobenzoate (619-42-1, Sigma 407593-10G), followed by (2) double cyanation (CuCN in DMSO, 150 °C under Ar), using published procedures.[3] The two phthalonitrile precursors were reacted together, using literature procedures ($ZnCl_2$ and 1,5-diazabiciclo(5.4.0)undec-7-ene, in *n*-pentanol at 145 °C for 24 h), to obtain the tri-*tert*-butyl ester Zinc phthalocyanine mono ester. The ester was deprotected to acid using literature procedure.[4] The final characterization of the acid phthalocyanine was in accordance to literature procedure.[5]

## 2. Apparatus and Characterization

Size exclusion chromatography (SEC) was carried out using Sephadex™ LH-20 as stationary phase and methanol as mobile phase.

Fourier-transform infrared spectra (KBr) were recorded on a Perkin Elmer 2000 spectrometer.

X-ray photoemission spectroscopy (XPS) spectra were measured on a SPECS Sage HR 100 spectrometer using a non-monochromatised Mg Kα radiation of 1253.6 eV and 252 W, in an ultra-high vacuum chamber at pressure below 8 × $10^{-8}$ mbar. The calibration was done using the $3d_{5/2}$ line of Ag (FWHM 1.1 eV). All spectra were collected with pass energy of 15 eV and 0.15 eV/step, respectively. For each analysis, an aqueous solution (ca. 3 mg/mL) of material were deposited on a gold thin film. Avantage (Thermo Fisher Scientific) software was used for data processing and fitting. For charge corrections the C 1s (C-C/C=C peak at 284.8 eV) was used as reference. Curve fittings of the C 1s and N 1s spectra were realized using a Gaussian-Lorentzian peak shape after performing a Shirley background correction, to finally obtain the relative percentage of each type of bond inside the analyzed sample. TGA analyses were performed using a TA Instruments TGA Q500 with a ramp of 10 °C/min under $N_2$ from 100 to 700 °C.

Atomic force microscopy (AFM) images were recorded using a Nanoscope IIIa, VEECO Instruments. As a general procedure to perform AFM analyses, tapping mode with a HQ:NSC19/Al BS probe (65 kHz; 0.6 N $m^{-1}$) (MikroMasch) from drop cast of samples in a methanolic solution (concentration of few mg $mL^{-1}$) on a mica substrate was performed. The obtained AFM-images were analyzed in Gwyddion 2.35.

Ultrafast transient absorption (TA) experiments were conducted using an amplified Ti/sapphire laser system (Clark MXR CPA2101 and 2110, FWHM = 150 fs, $\lambda_{exc}$ = 387 or 675 nm, 200-300 nJ per pulse) with TA pump / probe Helios detection systems from Ultrafast Systems. White light was generated using a sapphire crystal. Optical densities (OD) of the samples were around 0.5 at the excitation wavelengths. A magic angle configuration was employed to avoid rotational dynamics.

Electrochemistry was performed with NCND-ZnPc, as well as NCNDs and ZnPc references. The compounds were dissolved in methanol (MeOH) with addition of 0.2 M of electrolyte tetrabutylammonium hexafluorophosphate ($TBAPF_6$). Potentials were measured against an $Ag/AgNO_3$ reference electrode, while ferrocene was used as external standard. Prior to any measurements, the solutions (2-5 mL) were flushed with Argon for at least 20 minutes. Both were investigated by Squarewave Voltammetry within a window of approximately 2.6 V (−1.3 V to +1.3 V vs $Ag/AgNO_3$) and without showing any signs of solvent electrolysis.

## 3. Synthesis of NCND-ZnPc

In a two necked round bottom flask, ZnPc (12.4 mg, 15.8 μmol) was dissolved in anhydrous DMF (4.0 mL) under Ar, followed by addition of EDC·HCl (6.1 mg, 31.6 μmol) and NHS (3.6 mg, 31.6 μmol). The whole mixture was left stirring under Ar atmosphere at r.t. for 30 minutes. NCNDs (7.0 mg) were subsequently added as solids, under Argon, and the reaction was left stirring at the same conditions for 18 hours. Then the solvent was removed under reduced pressure. The blue residue was dissolved in methanol and purified by SEC. The methanolic fractions were collected and dried under vacuum, then dissolved in water and lyophilized affording 15.43 mg of a blue solid.

## 4. Supporting Figures

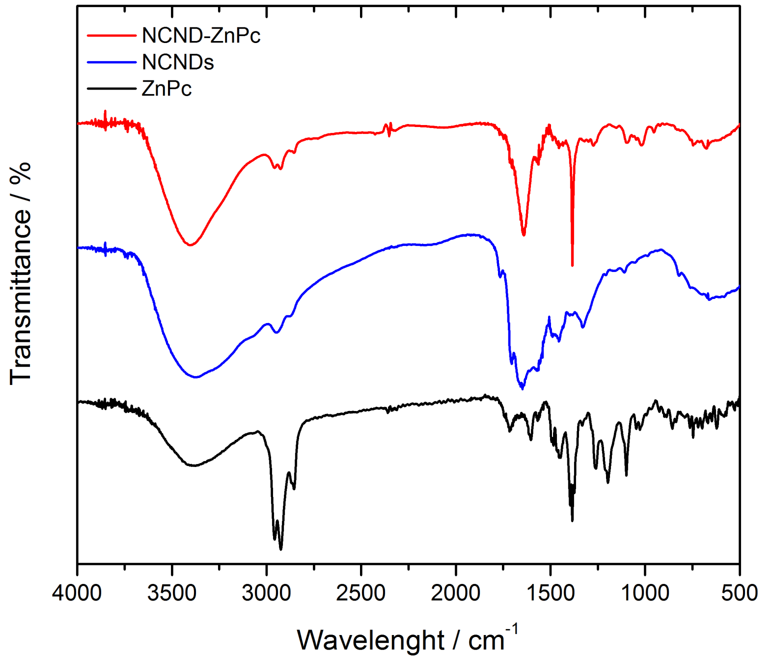


**Figure S1** FT-IR spectra of NCND-ZnPcs (black), NCNDs (red) and ZnPcs (blue).

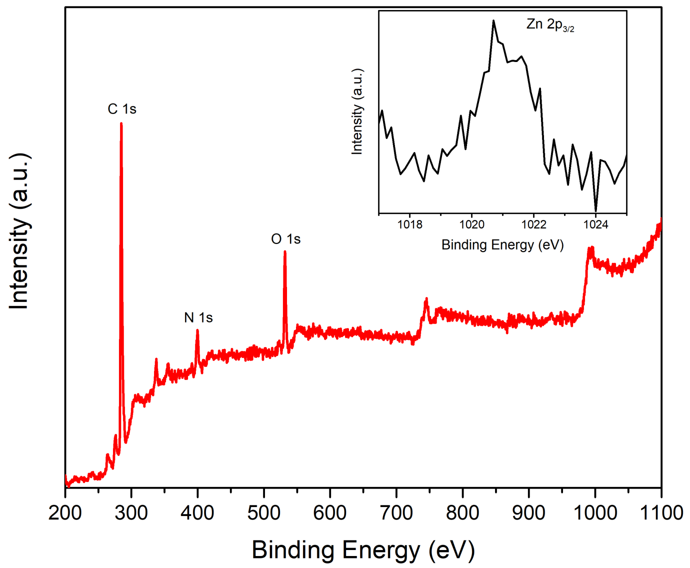


**Figure S2** XPS survey spectrum of NCND-ZnPc showing C 1s, N 1s, O 1s. The inset shows the high-resolution spectrum of Zn $2p_{3/2}$.

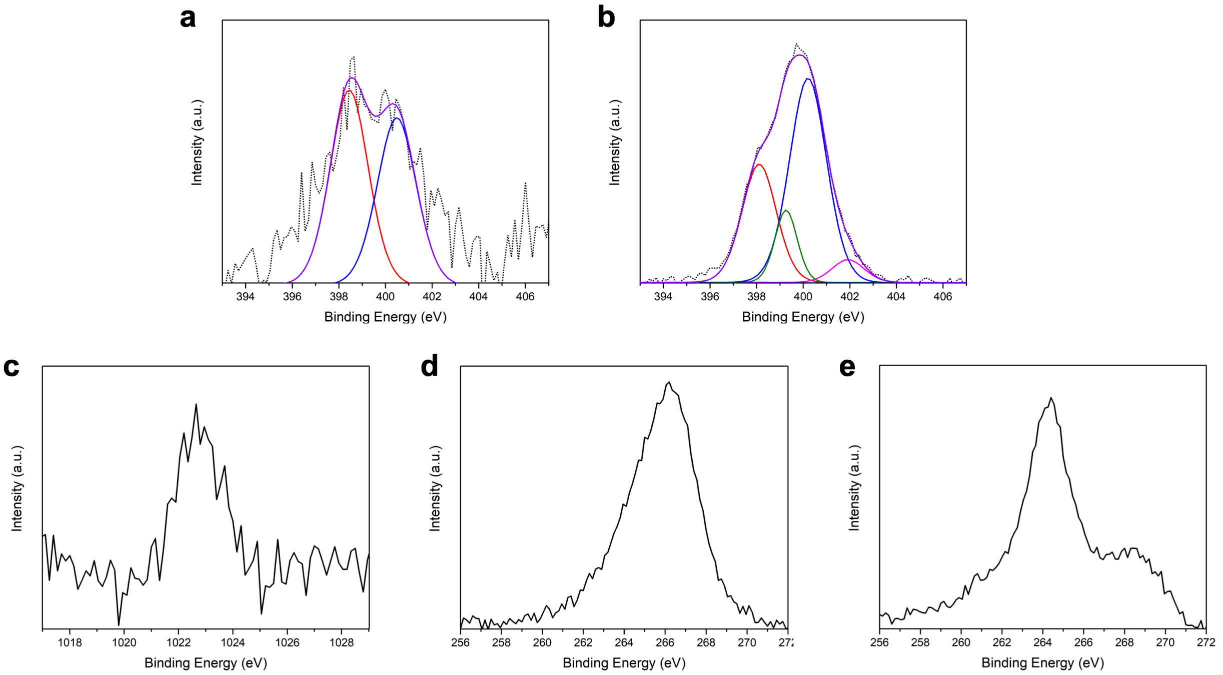


**Figure S3** (a) N 1s spectrum of ZnPc deconvoluted into two peaks (398.4, 400.5 eV) corresponding to C=N and nitrogen coordinated to Zn. (b) N 1s spectrum of NCND-ZnPc deconvoluted into four peaks (398.1, 399.3, 400.2, 401.9 eV) corresponding to nitrogen coordinated to Zn, C=N, C-N, $NH_2$ and N-$C_3$. The percentage of $NH_2$ calculated from the peak at 399.3 eV (12%) is lower than the one reported for NCNDs alone (35 %).[1] (c) Zn $2p_{3/2}$ spectrum of ZnPc. Zn Auger LMM of (d) ZnPc and (e) NCND-ZnPc.

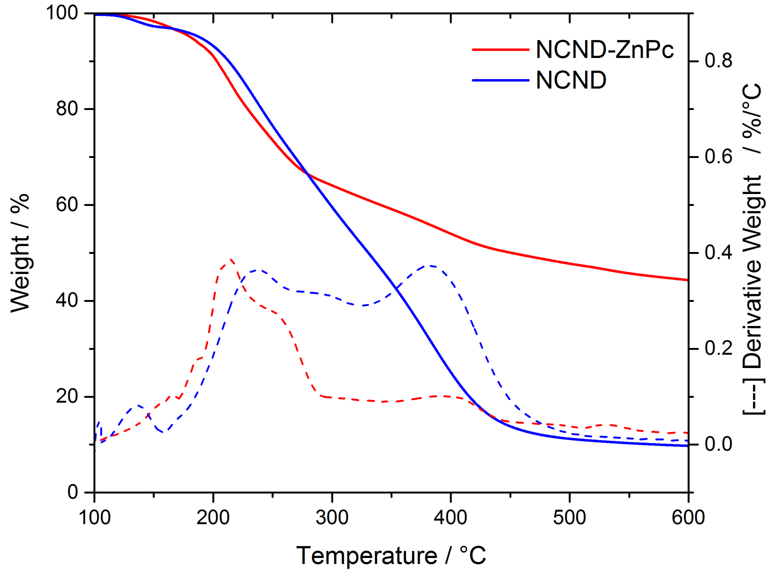


**Figure S4** TGA analyses of NCND (blue line) and NCND-ZnPc (red line). The dashed lines represent the 1st derivatives of the weight loss.

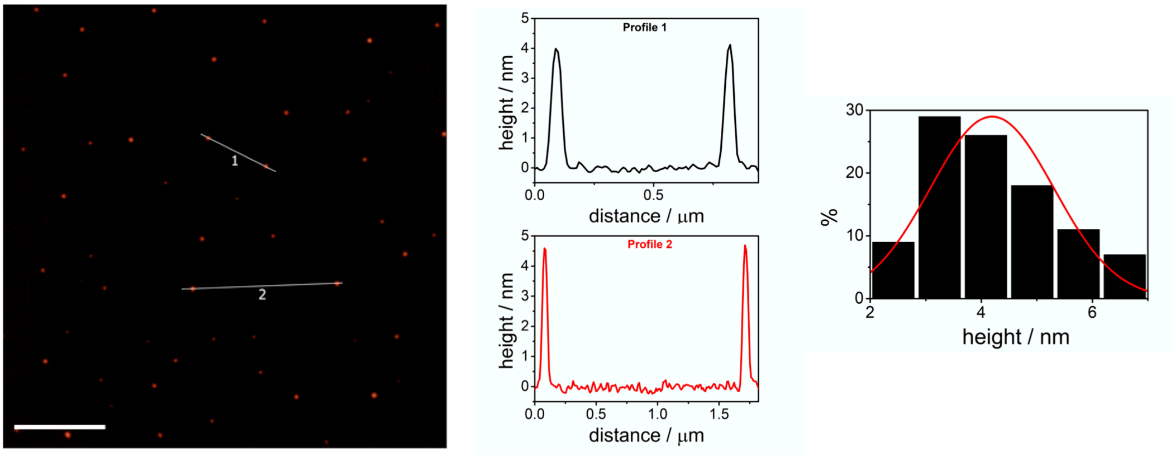


**Figure S5** 2D tapping mode AFM images (5.0 × 5.0 µm) from drop-cast NCND-ZnPc methanolic solutions on mica substrates (left); height profile along the dashed line (center); height sizes distribution histogram and superimposed Gaussian fit (right).

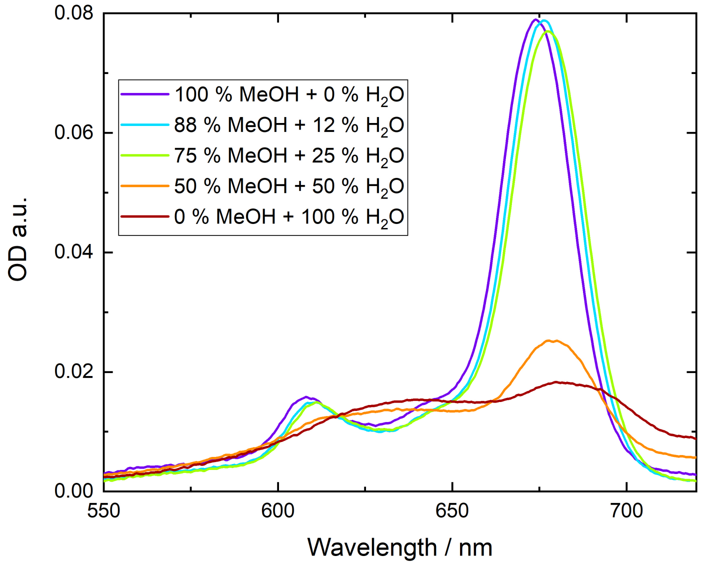


**Figure S6** Aggregation experiments of ZnPc. Different amounts of water are added to a solution of ZnPc in methanol, at room temperature.

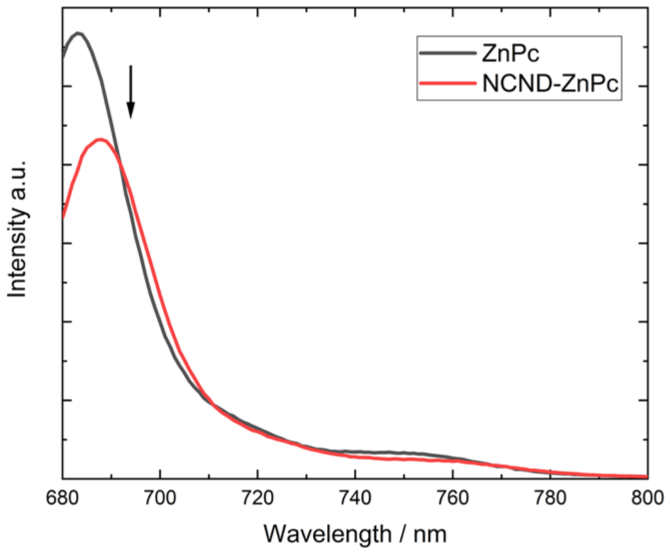


**Figure S7** Emission spectra of matching absorbance solutions of ZnPc and NCND-ZnPc under 675 nm excitation in methanol at room temperature.

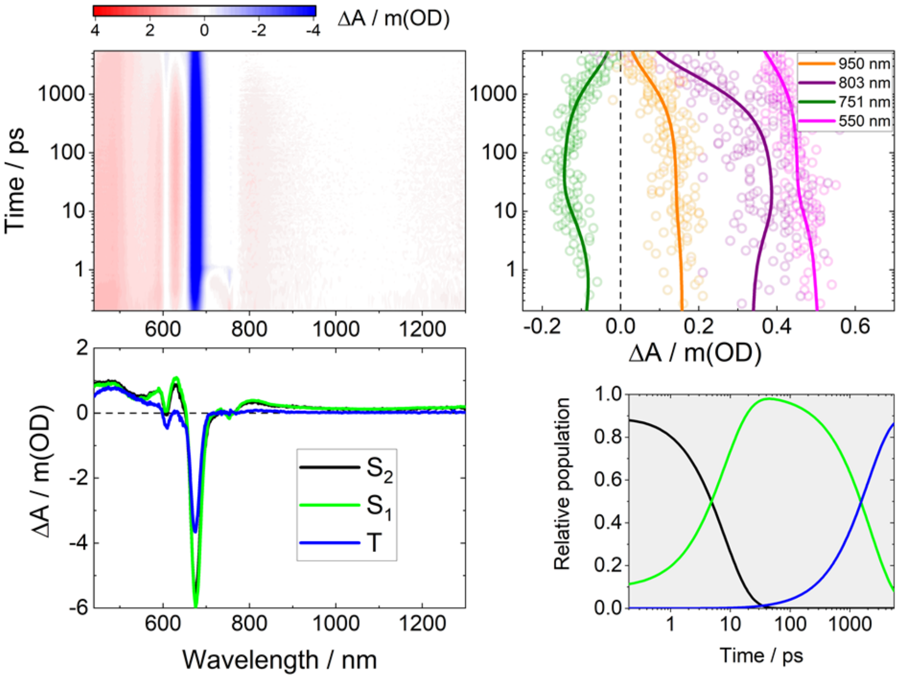


**Figure S8** Top left: Differential absorption 3D map obtained upon fsTAS on ZnPc in methanol at room temperature upon 387 nm excitation. Top right: Time absorption profiles and corresponding fittings at 950 (orange), 803 (purple), 751 (dark green) and 550 (magenta) nm. Bottom left: Species associated differential spectra of the $S_2$ (black), $S_1$ (green) and T (blue) excited states. Bottom right: concentration evolution over time.

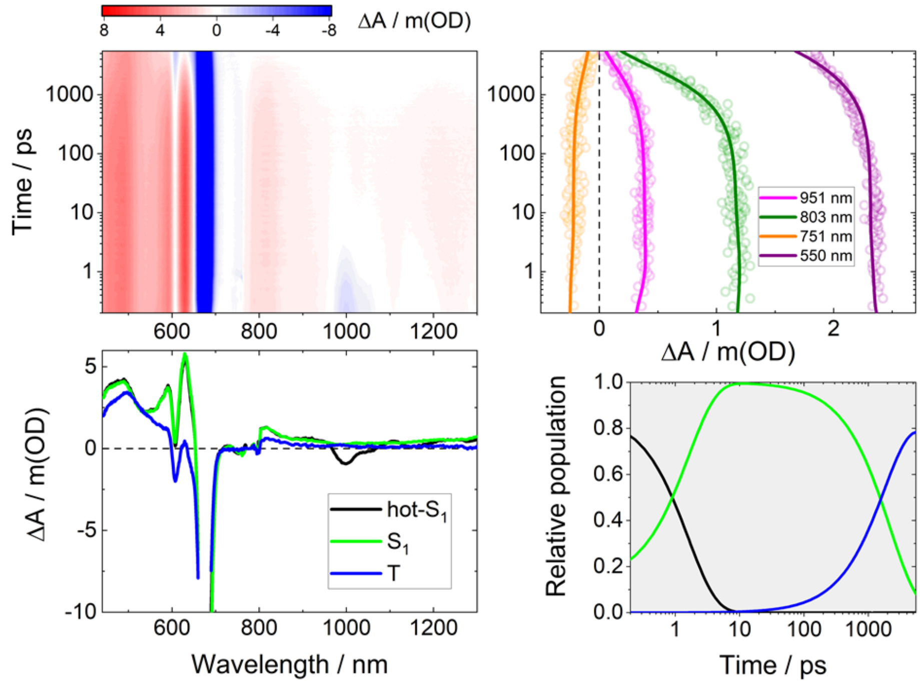


**Figure S9** Top left: differential absorption 3D map obtained upon fsTAS on ZnPc in methanol at room temperature upon 675 nm excitation. Top right: Time absorption profiles and corresponding fittings at 951 (magenta), 803 (dark green), 751 (orange) and 550 (purple) nm. Bottom left: Species associated differential spectra of the hot-$S_1$ (black), $S_1$ (green) and T (blue) excited states. Bottom right: concentration evolution over time.

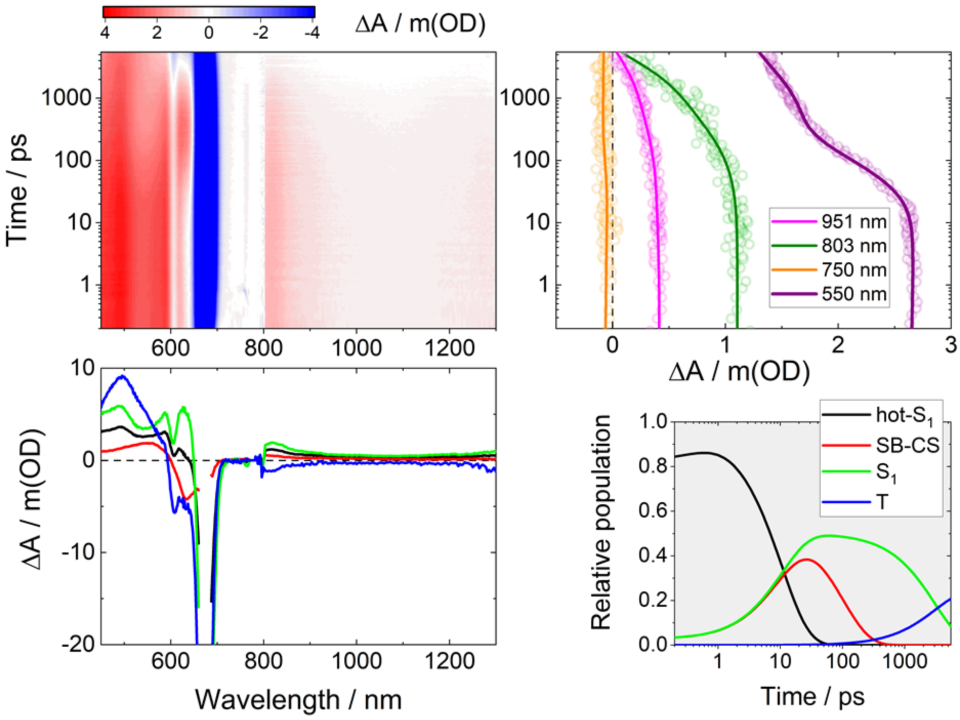


**Figure S10** Top left: Differential absorption 3D map obtained upon fsTAS on NCND-ZnPc in methanol at room temperature upon 675 nm excitation. Top right: Time absorption profiles and corresponding fittings at 951 (magenta), 803 (dark green), 750 (orange) and 550 (purple) nm. Bottom left: Species associated differential spectra of the hot-$S_1$ (black), SB-CS (red), $S_1$ (green) and T (blue) excited states. Bottom right: concentration evolution over time.

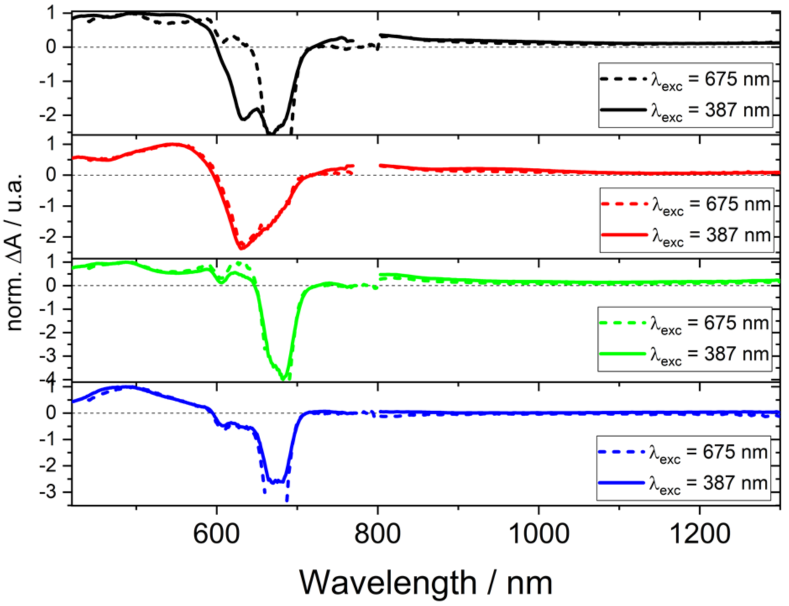


**Figure S11** Species associated differential spectra for NCND-ZnPc in methanol at room temperature under 387 (solid curves) and 675 (dashed curves) nm excitation. From top to bottom: SB-CT / hot-$S_1$ (black), SB-CS (red), $S_1$ (green), T (blue).

## 5. References


1. Arcudi, F.; Đorđević, L.; Prato, M., Synthesis, Separation, and Characterization of Small and Highly Fluorescent Nitrogen-Doped Carbon NanoDots. *Angew. Chem. Int. Ed.* **2016,** *55* (6), 2107-2112.
2. Đorđević, L.; Arcudi, F.; Prato, M., Preparation, functionalization and characterization of engineered carbon nanodots. *Nat. Protoc.* **2019,** *14* (10), 2931-2953.
3. Dieing, R.; Schmid, G.; Witke, E.; Feucht, C.; Dreßen, M.; Pohmer, J.; Hanack, M., Soluble Substituted μ-Oxo(phthalocyaninato)iron(III) Dimers. *Chem. Ber.* **1995,** *128* (6), 589-598; Tilley, J. W.; Clader, J. W.; Zawoiski, S.; Wirkus, M.; LeMahieu, R. A.; O'Donnell, M.; Crowley, H.; Welton, A. F., Biphenylcarboxamide derivatives as antagonists of platelet-activating factor. *J. Med. Chem.* **1989,** *32* (8), 1814-20.
4. Arcudi, F.; Strauss, V.; Đorđević, L.; Cadranel, A.; Guldi, D. M.; Prato, M., Porphyrin Antennas on Carbon Nanodots: Excited State Energy and Electron Transduction. *Angew. Chem. Int. Ed.* **2017,** *56* (40), 12097-12101.
5. Cid, J.-J.; Yum, J.-H.; Jang, S.-R.; Nazeeruddin, M. K.; Martínez-Ferrero, E.; Palomares, E.; Ko, J.; Grätzel, M.; Torres, T., Molecular Cosensitization for Efficient Panchromatic Dye-Sensitized Solar Cells. *Angew. Chem. Int. Ed.* **2007,** *46* (44), 8358-8362.
